# Evidence for Decreased Density of Calretinin-Immunopositive Neurons in the Caudate Nucleus in Patients With Schizophrenia

**DOI:** 10.3389/fnana.2020.581685

**Published:** 2020-11-13

**Authors:** Istvan Adorjan, Bin Sun, Virginia Feher, Teadora Tyler, Daniel Veres, Steven A. Chance, Francis G. Szele

**Affiliations:** ^1^Department of Physiology, Anatomy and Genetics, University of Oxford, Oxford, United Kingdom; ^2^Nuffield Department of Clinical Neuroscience, University of Oxford, Oxford, United Kingdom; ^3^Department of Anatomy, Histology and Embryology, Semmelweis University, Budapest, Hungary; ^4^Institute of Clinical Sciences, Imperial College London, London, United Kingdom; ^5^Medical Research Council (MRC) London Institute of Medical Sciences, London, United Kingdom; ^6^Department of Biophysics and Radiation Biology, Semmelweis University, Budapest, Hungary

**Keywords:** schizophrenia, caudate nucleus, interneuron, calretinin, neuropeptide Y

## Abstract

Schizophrenia (SCH) and autism spectrum disorder (ASD) share several common aetiological and symptomatic features suggesting they may be included in a common spectrum. For example, recent results suggest that excitatory/inhibitory imbalance is relevant in the etiology of SCH and ASD. Numerous studies have investigated this imbalance in regions like the ventromedial and dorsolateral prefrontal cortex (DLPFC). However, relatively little is known about neuroanatomical changes that could reduce inhibition in subcortical structures, such as the caudate nucleus (CN), in neuropsychiatric disorders. We recently showed a significant decrease in calretinin-immunopositive (CR-ip) interneuronal density in the CN of patients with ASD without significant change in the density of neuropeptide Y-immunopositive (NPY-ip) neurons. These subtypes together constitute more than 50% of caudate interneurons and are likely necessary for maintaining excitatory/inhibitory balance. Consequently, and since SCH and ASD share characteristic features, here we tested the hypothesis, that the density of CR-ip neurons in the CN is decreased in patients with SCH. We used immunohistochemistry and qPCR for CR and NPY in six patients with schizophrenia and six control subjects. As expected, small, medium and large CR-ip interneurons were detected in the CN. We found a 38% decrease in the density of all CR-ip interneurons (*P* < 0.01) that was driven by the loss of the small CR-ip interneurons (*P* < 0.01) in patients with SCH. The densities of the large CR-ip and of the NPY-ip interneurons were not significantly altered. The lower density detected could have been due to inflammation-induced degeneration. However, the state of microglial activation assessed by quantification of ionized calcium-binding adapter molecule 1 (Iba1)- and transmembrane protein 119 (TMEM119)-immunopositive cells showed no significant difference between patients with SCH and controls. Our results warrant further studies focussing on the role of CR-ip neurons and on the striatum being a possible hub for information selection and regulation of associative cortical fields whose function have been altered in SCH.

## Introduction

Schizophrenia (SCH) is a chronic and serious mental illness which puts an enormous burden on the individual, families, and society. Recent meta-analyses based on multinational surveys and robust longitudinal studies established the lifetime and 1-year prevalence of SCH at 0.4% (Saha et al., 2005) and 1.1% (Regier et al., 1993), respectively. The condition has multiple genetic risk factors (Fromer et al., 2014; Ripke et al., 2014; Pardiñas et al., 2018), possibly interplaying with several environmental risk factors (Zwicker et al., 2018). However, the neuropathology of SCH is still unclear and much remains to be discovered about the neuroanatomical correlates and causes of SCH.

At the functional level, excitatory/inhibitory imbalance in the brain has been implicated in SCH (Gao and Penzes, 2015; Yang and Tsai, 2017), and may lead to positive symptoms such as hallucinations (Jardri et al., 2016), and to negative or cognitive symptoms (Kehrer et al., 2008). The vast majority of studies in SCH have focussed on the cerebral cortex while the basal ganglia are comparatively under-investigated. However, several indications point to the caudate nucleus (CN) as being a primary node of dysfunction in SCH (Li et al., 2018). For instance, functional hypo-connectivity was found between the frontal cerebral cortex and the CN in treatment-naïve first-episode patients with SCH (Lin et al., 2018). Furthermore, reduced structural connectivity was observed between the frontal cerebral cortex and the CN in SCH (Levitt et al., 2017).

In our current study we primarily aimed to investigate the involvement of striatal interneurons in SCH. We focussed on the CN and two major interneuron populations, calretinin-immunopositive (CR-ip) and neuropeptide Y-immunopositive (NPY-ip) neurons which together constitute more than 50% of CN interneurons (Wu and Parent, 2000; Petryszyn et al., 2014), and approximately 10% of all CN neurons in human (Graveland and DiFiglia, 1985), whereas in rodents they may only be around 0.5% (Rymar et al., 2004). Our work provides strong evidence for the density of CR-ip interneurons being significantly decreased in the CN of patients with SCH and highlights the potential importance of this specific interneuron population in SCH pathophysiology. These results are very similar to autism spectrum disorder (ASD; Adorjan et al., 2017) and further suggest these neuropsychiatric disorders may be on a continuum. Our work also predicts an excitatory/inhibitory imbalance in the CN and warrants further studies focussing on the role of CR-ip neurons.

## Materials and Methods

### Subjects

Tissue from subjects was provided by the Netherlands Brain Bank (NBB) Netherlands Institute for Neuroscience, Amsterdam (open access^1^) and the Oxford Brain Bank (OBB). Eight cases with clinical diagnosis of SCH (based on DSM III and DSM IV) and six control subjects without history of psychiatric disorders were studied. All material was collected from donors from whom written informed consent had been obtained by the OBB or NBB for brain autopsy and use of material and clinical information for research purposes. The demographic characteristics of the cohort are shown in Table 1, Supplementary Table S1. The mean values of age, gender, post-mortem interval and time in paraformaldehyde were not significantly different in the diagnostic groups examined (Table 1 and Supplementary Table S2). Sampling was done by assistants of the OBB and NBB supervised by trained neuropathologists. Depending on availability of tissue blocks from subjects with SCH, regions were selected containing the head of the CN between 12.5 and 1 mm rostral to the anterior commissure according to the Human Brain Atlas (Mai et al., 2008, Supplementary Figure S1). Corresponding levels of the head of the CN were chosen from control subjects. No statistically significant difference was observed between the diagnostic groups regarding levels of sampling (SCH: −4.25mm ± 0.55, CTR: −6.96mm ± 1.31, *n* = 12, *p* = 0.10, measured from the anterior commissure). Tissue blocks were also requested from the dorsolateral prefrontal cortex (DLPFC; Brodmann area 9) and paraffin embedded tissue was provided from the medial frontal gyrus. Fresh-frozen tissue from the superior frontal gyrus was used for qPCR experiments.

**TABLE 1:** Main demographic characteristics of controls and patients with schizophrenia (SCH).

Identifier	Diagnosis	Age	Gender	PMI (h)	Cause of death	Regions investigated
#1	Control	57	F	7	Urothelium carcinoma, euthanasia	CN, BA9
#2	Control	60	F	8	Septicaemia	CN, BA9
#3	Control	78	F	5	Bronchopneumonia	CN, BA9
#4	Control	70	F	6	Pulmonary carcinoma	CN, BA9
#5	Control	55	M	8	Oesophageal cancer, euthanasia	CN, BA9
#6	Control	55	M	7	A. mes. sup. thrombosis	CN, BA9
#7	SCH	66	F	11	Pancreas carcinoma	CN, BA9
#8	SCH	64	M	19	Pulmonary embolism	CN, BA9
#9	SCH	79	F	5	Heart failure	CN, BA9
#10	SCH	55	F	10	Suicide	CN, BA9
#11	SCH	63	F	5	Breast carcinoma	CN*
#12	SCH	92	F	8	Pneumonia	BA9*
#13	SCH	59	M	13	Coronary insufficiency	BA9*
#14	SCH	50	M	34	Bleeding from oesophageal varix	CN*

CN, caudate nucleus; BA9, Brodmann area 9; *only one region was available from these cases.

### Immunohistochemistry

Serial sections (6-μm thick) were cut in the coronal plane from paraffin-embedded blocks and mounted on slides. Consecutive sections were stained for the four antigens investigated. Our immunohistochemical analysis was done as described in detail in earlier studies (Adorjan et al., 2017, 2019). Briefly, the sections were dewaxed through a graded alcohol series and treated with 3% H_2_O_2_ solution (in phosphate buffered saline, pH 7.4) for 30 min. Antigen retrieval was applied by autoclaving the slides in citrate buffer (0.01 M, pH 6.0) at 121°C for 10 min. The following primary antibodies were used: anti-calretinin (rabbit, 1:300, Chemicon, AB5054), anti-neuropeptide Y (rabbit, 1:250, Abcam, ab30914), anti ionized calcium-binding adapter molecule 1 (Iba1; rabbit, 1:500, WAKO, 01919741) and anti-transmembrane protein 119 (TMEM119; rabbit, 1:200, Abcam, ab185333) in Tris-buffered saline/Triton ^TM^ X-100 (pH 7.4) for 1 h (100 μl/section). Sections were then incubated with horseradish peroxidase-linked secondary antibody from the Envision Kit (Dako, K-5007) for 1 h (100 μl/section) and labeling was visualized by DAB from the same Envision Kit applied for 90 s (100 μl/section). During incubation with primary and secondary antibodies slides were put into Sequenza System coverplates and rack (Thermo Scientific, 72110017, 73310017). Two rinses with Tris-buffered saline/Triton ^TM^ X-100 (pH 7.4) were applied between the above-described steps of immunohistochemistry (1,000 μl each). Haematoxylin nuclear counterstain was applied for 20 s. Sections were dehydrated through a graded alcohol series and coverslipped with DePeX. No labeling was observed when primary antibodies were omitted from the protocol.

### Image Analysis, Quantification

Sections were digitized using a slide scanner (Aperio ScanScope AT Turbo, Leica Biosystems) at 20× magnification and stored on a server (msdlt-slide.dpag.ox.ac.uk). The regions of interest (the whole CN and ∼2 mm × 1 mm columns with all cortical layers) were outlined using the ImageScope program (Aperio, v11.2.0.780, Supplementary Figure S2). Cross-sectional areas analyzed in this study are shown in Supplementary Table S3. The longest diameter of every CR- and NPY-ip cell body in the sections was manually measured as described in Adorjan et al. (2017). Three investigators contributed to the quantification and all were blinded to the diagnoses of the subjects through random coding of the subject identifiers. In contrast to many other brain diseases where pathology is histologically evident, there were no major histopathological patterns recognizable in cases with SCH based on our histochemical stainings, and it was not possible to determine which subjects were controls versus patients with SCH. Neuronal cell bodies with a diameter > 6 μm and a width > 2 μm were included in further statistical analysis in concert with our previous study (Adorjan et al., 2017). For Iba1- and TMEM119-immunohistochemistry, the stained area fraction was obtained by the Aperio Positive Pixel Count Algorithm (parameters are shown in Supplementary Table S4) in line with (Adorjan et al., 2017).

### Reverse-Transcriptase Quantitative PCR Analysis

Cortical tissue (superior frontal gyrus) was dissected from individual samples and stored at −80°C. Total RNA was extracted with RNeasy Mini Kit (Qiagen) following the manufacturer’s instructions, with genomic DNA removed by on-column DNase set (Qiagen). SuperScript III RT kit (Qiagen) was used for reverse transcription and the cDNA was then subjected to real-time quantitative PCR (StepOnePlus, Applied Biosystems) with SYBR Green PCR Master Mix (Kapa Biosystems). Technical duplicates were included for all reactions. To select the internal references, seven candidate genes (GAPDH, PPIA, ACTB, SDHA, SNCA, TBP, UBC) were analyzed with NormFinder (Andersen et al., 2004; Weickert et al., 2010; Dean et al., 2016). The combination of GAPDH and PPIA had the best stability value (0.00287) and was used to normalize the target gene levels. The relative expression was calculated and presented as 2^–ΔΔ*Ct*^. The primer sequences are listed in Supplementary Table S5. Quantitative PCR experiments were performed with technical duplicates.

### Statistical Analyses

Results are presented as means ± standard error of the mean. One section per subject was used per immunostain (Supplementary Table S3). Statistical analyses were performed using the SPSS software (version 22.0). Student’s *t*-test (unpaired, two-tailed) was used to assess if the means of variables between the two diagnostic groups were significantly different. Fisher’s exact test was used to compare the distribution of gender between the groups. For correlation analyses Pearson’s correlation test was applied. Results of statistical comparisons presented are all based on unpaired, two-tailed Student’s *t*-tests if not stated otherwise.

In order to control for demographic variables and to reveal which neuronal subtype was the most affected in the CN, the univariate analysis of variance [general linear model (GLM)] was applied. The densities of the three subtypes (small, medium and large CR-ip neurons) and the total CR-ip population were tested separately. The densities of NPY-ip neurons, stained area fractions of Iba1 and TMEM119 and mRNA levels were also tested as dependent variables in separate univariate analyses of variance. Diagnosis was tested as a between-subjects factor and potential confounders (PMI, age, gender, time in PFA) as covariates. Significance level was set to 5% (α = 0.05).

## Results

### Three Types of Calretinin-Immunopositive Neurons Are Present in the Control Human Caudate Nucleus Based on Morphological Classification

**FIGURE 1:**
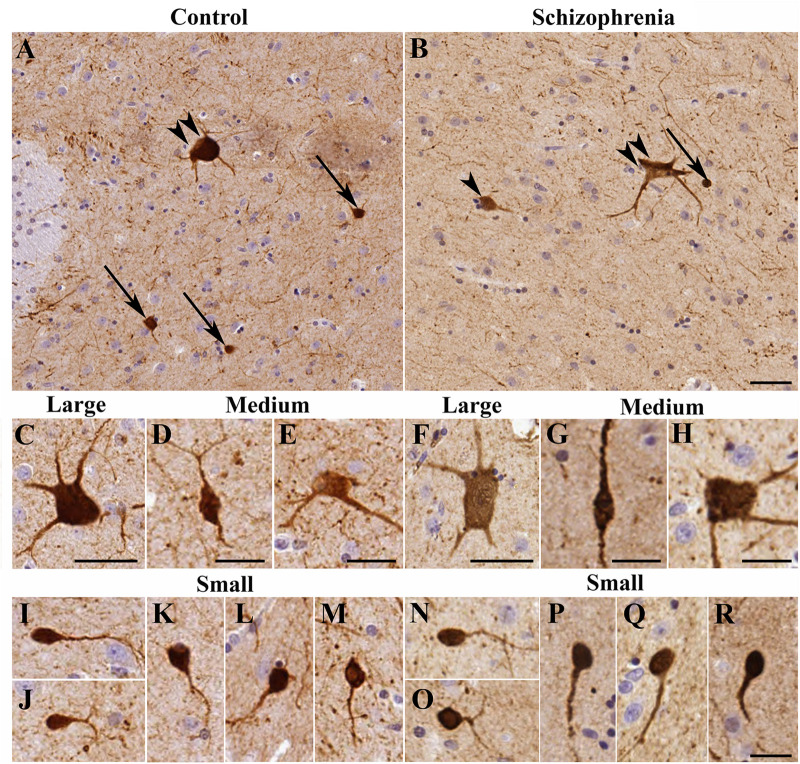
The morphology of calretinin-immunopositive (CR-ip) neurons in the caudate nucleus (CN) was similar in controls and cases with schizophrenia (SCH). Images are shown from 4 controls and 4 subjects with SCH. Representative viewfields from a control case **(A)** and from a case with SCH **(B)**; double arrowhead – large neuron, arrowhead – medium neuron, arrow – small neuron. **(C)** Large neuron from a control subject. **(D)** Medium neuron with bipolar morphology from a control case. **(E)** Multipolar medium neuron from a control case. **(F)** Large neuron from a case with SCH. **(G,H)** Medium neurons from cases with SCH. **(I–M)** Small neurons from control cases. **(N–R)** Small CR-ip neurons from subjects with SCH. Scale bars: **(D,E,G,H)** 20 μm; **(I–R)**: 15 μm; otherwise 40 μm.

Morphological assessments were carried out based on the shape and diameters of 14,092 CR-ip neurons in controls (*N* = 6). Three types of CR-ip neurons were found in the human CN in line with (Petryszyn et al., 2014) and our earlier study (Adorjan et al., 2017; Figure 1A). In brief, the small CR-ip neurons in controls (11,474 neurons measured) had round or oval perikarya and their diameter ranged from 6 to 15 μm (Figures 1I–M). Their average diameter was 9.43 ± 0.13 μm. The small CR-ip neurons usually had one process in the plane of the section (Figures 1I–M). The medium-sized CR-ip neurons in controls had multipolar or bipolar perikarya (with multipolar dominance) and two to three processes (Figures 1D,E). Their diameter ranged from 15 to 25 μm with an average of 19.20 ± 0.14 μm (1,570 neurons measured). The large neurons in controls had multipolar perikarya with three to five processes in the plane of the section (Figure 1C). Their diameter ranged from 25 to 60 μm with an average of 31.24 ± 0.54 μm (1,048 neurons measured).

### Diameter and Ratios of Calretinin-Immunopositive Neuronal Subtypes in the Caudate Nucleus Are Unchanged in Schizophrenia

We next compared the control CR-ip caudate neurons with 7,351 CR-ip neurons in patients with SCH (*N* = 6). We analyzed every caudate nucleus CR-ip neuron in our sections. Qualitative comparison of process numbers, branching and somal shape did not reveal conspicuous differences between the control and SCH groups (Figures 1B,F–H,N–R). No significant differences were found between controls and subjects with SCH in terms of the average diameters of the CR-ip neurons either. The diameters of the CR-ip populations were as follows: small – 9.37 ± 0.17 μm (5,810 neurons measured, *n* = 12, *p* = 0.803 compared to controls), medium – 19.20 ± 0.14 μm (915 neurons, *n* = 12, *p* = 0.975 compared to controls), large – 31.27 ± 0.25 μm (625 neurons, *n* = 12, *p* = 0.962 compared to controls). The ratios of different CR-ip subtypes (small, medium and large) were also very similar in both groups (controls: 81.61, 11.09, and 7.29%; SCH: 80.01, 12.09, and 7.88%, *P* = 0.382, 0.299, and 0.598, respectively).

To evaluate the efficacy of measuring diameters instead of neuronal cross-sectional areas we investigated the correlation of these two variables by measuring 60 randomly selected neurons from each case (altogether 720 neurons). Similar to diameters, cross-sectional areas were not significantly different between the diagnostic groups: controls – 118.64 ± 3.12 μm^2^, SCH – 125.28 ± 4.55 μm^2^, *n* = 12, *p* = 0.259. The proportion of the small, medium and large subtypes measured in this analysis were: controls: 84.44, 10.00, and 5.55%; SCH: 85.83, 9.72, and 4.44%, *n* = 12, *p* = 0.608, 0.897, and 0.334, respectively. There was a very strong correlation between diameters and cross-sectional areas in controls (*r* = 0.92, *p* < 0.0001, Pearson’s correlation) and subjects with SCH (*r* = 0.95, *p* < 0.0001, Pearson’s correlation) that underlined the feasibility of the diameter measurements and compatibility of the two approaches.

### The Density of Calretinin-Immunopositive Neurons in the Caudate Nucleus Is Significantly Lower in Schizophrenia

**FIGURE 2:**
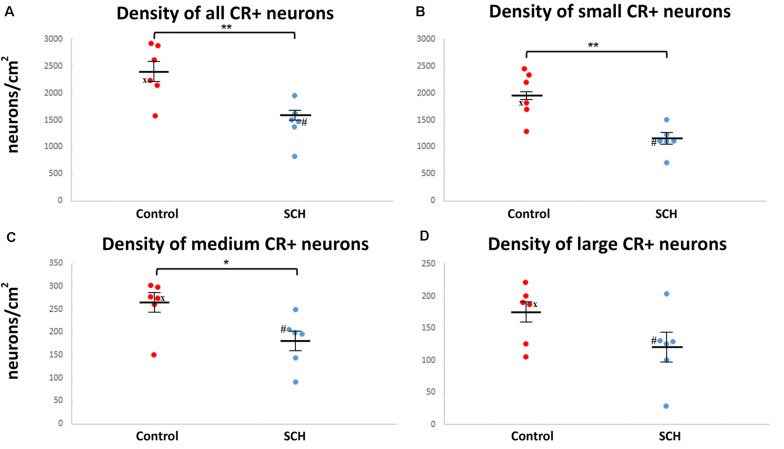
The density of caudate calretinin-immunopositive (CR-ip) neurons is lower in subjects with schizophrenia (SCH). Graphs showing the number of CR-ip neurons per square cm as a total population **(A)** and subdivided into the small **(B)**, medium **(C)** and large **(D)** diameter populations in controls and cases with SCH. *p < 0.05, **p < 0.01. ‘*x*’ labels a control case (#5) only received anti-psychotic medication as part of the palliative sedation treatment before euthanasia. ‘#’ labels a case with schizophrenia (#8) received minimal anti-psychotic medication.

There was a 39% decrease in the density of small CR-ip neurons in subjects with SCH compared to controls (SCH: 1,178 ± 108 neurons/cm^2^, controls: 1,944 ± 183 neurons/cm^2^, *n* = 12, *p* < 0.01, Figure 2B). Moreover there was a significant 31% decrease in the density of medium CR-ip neurons in the SCH group (SCH: 181 ± 23 neurons/cm^2^, controls: 262 ± 23 neurons/cm^2^, *n* = 12, *p* < 0.05, Figure 2C). Regarding the large CR-ip neurons, a 28% lower density was detected in the SCH group, however this was not statistically significant (SCH: 123 ± 29 neurons/cm^2^, controls: 172 ± 18 neurons/cm^2^, *n* = 12, *p* = 0.131, Figure 2D). When the three CR-ip subpopulations were taken together, the SCH group showed a statistically significant 38% lower density (SCH: 1,482 ± 151 neurons/cm^2^, controls: 2,378 ± 210 neurons/cm^2^, *n* = 12, *p* < 0.01, Figure 2A).

In order to reveal which subtype was the most affected in the CN, the General Linear Model univariate analysis of variance (GLM) was applied. The three subtypes (small, medium, and large CR-ip neurons) and the total CR-ip population were tested separately. Possible confounders such as age, gender and post-mortem-interval were taken into consideration and their potentially significant effect on the CR-ip neuronal densities was also tested. This analysis returned no significant effects of the aforementioned variables on the densities of CR-ip neurons (Table 2). On the contrary, only diagnosis was confirmed to have a significant effect on the density of the total CR-ip population (*n* = 12, *p* = 0.018, GLM, Table 2) which was driven by its effect on the density of the small CR-ip population (*n* = 12, *p* = 0.013, GLM, Table 2). Furthermore, there was no statistically significant correlation between age and the densities of different CR-ip subpopulations either in the CN or in the DLPFC (Supplementary Figure S3).

**TABLE 2:** General Linear Model univariate analysis of CR, NPY densities and stained area fraction of Iba1 and TMEM119 in caudate nucleus (CN) with PMI, age, and gender as covariates.

	Diagnosis	PMI	Age	Gender
TotalCR density	*p* = 0.018	*p* = 0.675	*p* = 0.862	*p* = 0.129
SmallCR density	*p* = 0.013	*p* = 0.847	*p* = 0.879	*p* = 0.110
MediumCR density	*p* = 0.102	*p* = 0.555	*p* = 0.692	*p* = 0.435
LargeCR density	*p* = 0.556	*p* = 0.166	*p* = 0.418	*p* = 0.500
NPY density	*p* = 0.062	*p* = 0.216	*p* = 0.487	*p* = 0.257
Iba1 SAF	*p* = 0.349	*p* = 0.078	*p* = 0.384	*p* = 0.313
TMEM119	*p* = 0.766	*p* = 0.205	*p* = 0.315	*p* = 0.920

*CR, calretinin; NPY, neuropeptide; Iba1, ionized calcium-binding adapter molecule 1; TMEM119, transmembrane protein 119.*

We next compared the CR-ip densities of subjects with SCH to controls (*n* = 11) from a previously published dataset (Adorjan et al., 2017) added to our current set of controls (*n* = 6). Because of the differences in terms of time of fixation in PFA and position of sampling between the cohorts, a GLM analysis was run with the following variables: age, gender, post-mortem interval, time of fixation and position of sampling. This analysis confirmed the significant effect of diagnosis on the density of the total CR-ip population (small, medium and large taken together) and particularly, on the density of the small CR-ip neurons (Supplementary Table S6). No significant effects of confounders on CR densities were seen except a positive effect of time in PFA on the density of the large CR-ip population (*n* = 23, *p* = 0.018, GLM, Supplementary Table S6).

### Caudate Nucleus Size Was Not Significantly Different Between Controls and Subjects With Schizophrenia

We compared the cross-sectional areas of the CN and found no significant difference between the diagnostic groups (controls: 0.99 ± 0.09 cm^2^, SCH: 0.90 ± 0.08 cm^2^, *n* = 11, *p* = 0.477). Cross-sectional areas of the CN were non-significantly smaller in subjects with SCH which if anything should have increased the density of CR-ip neurons. There was no statistically significant correlation between age and cross-sectional areas of the CN in the SCH group (*r* = 0.32, *p* = 0.59, Pearson’s correlation), in the CTR group (*r* = −0.37, *p* = 0.46, Pearson’s correlation), or even when these groups were merged (*r* = −0.14, *p* = 0.67, Pearson’s correlation). These data are shown as scatter plots in Supplementary Figure S4.

### Morphology and Density of NPY-Immunopositive Neurons in the Caudate Nucleus Were Not Significantly Different Between Controls and Subjects With Schizophrenia

**FIGURE 3:**
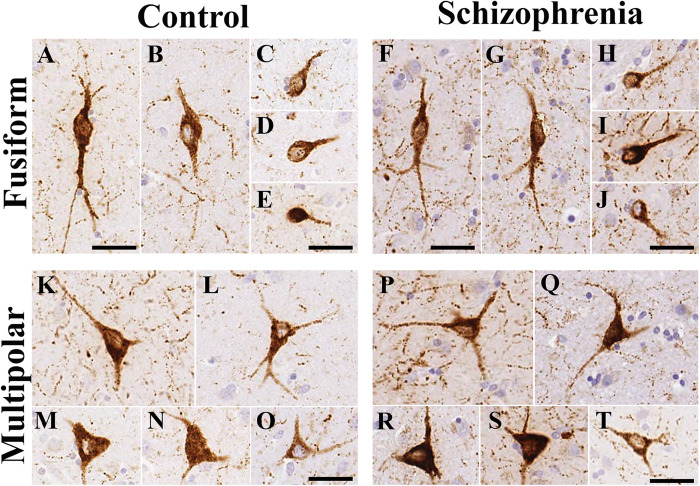
The morphology of neuropeptide Y-immunopositive (NPY-ip) neurons in the caudate nucleus (CN) was similar in controls and cases with schizophrenia (SCH). Images are shown from 4 controls and 4 subjects with SCH. **(A–E)** Fusiform NPY-ip neurons from control cases. **(F–J)** Fusiform NPY-ip neurons from cases with SCH. **(K–O)** Multipolar NPY-ip neurons from control subjects. **(P–T)** Multipolar NPY-ip neurons from cases with SCH. Scale bars: 30 μm.

In line with our previous study (Adorjan et al., 2017) there were two types of NPY-ip neurons in the CN based on their characteristic morphology (Figure 3). The fusiform-type neurons had round or oval perikarya usually with one or two primary processes in the plane of the section (Figure 3). The multipolar-type had pyramidal or multipolar perikarya usually with two to three processes. Both types of cell bodies ranged in diameter from 6 to 47 μm. There were no conspicuous morphological differences in the caudate nucleus NPY-ip neurons between the control and SCH groups.

**FIGURE 4:**
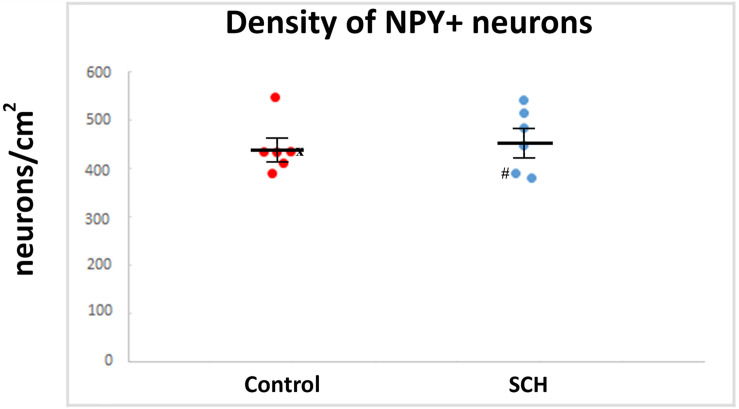
The density of neuropeptide Y-immunopositive (NPY-ip) neurons is not significantly different in cases with schizophrenia (SCH) compared to controls. Graph showing the number of NPY-ip neurons per square cm in controls and cases with SCH. ‘*x*’ labels a control case (#5) only received anti-psychotic medication as part of the palliative sedation treatment before euthanasia. ‘#’ labels a case with schizophrenia (#8) received minimal anti-psychotic medication.

We quantified every NPY-ip neuron present in the CN in our sections. Altogether, 910 neurons in the control group and 841 neurons in the SCH group were measured. No significant differences were found between the diagnostic groups in the average diameters of the NPY-ip neurons (controls: 14.03 ± 0.42 μm, SCH: 14.08 ± 0.79 μm, *n* = 12, *p* = 0.957). We found a 6% higher density of NPY-ip neurons in patients with SCH, however, this was not statistically significant (controls: 438 ± 23.83 cells/cm^2^, SCH: 462.83 ± 29.62 cells/cm^2^, *n* = 12, *p* = 0.506, Figure 4).

Similar to the lack of significant effect of diagnosis on the density of NPY-ip neurons (*n* = 12, *p* = 0.062, GLM, Table 2) there were no significant effects of other variables examined, such as post-mortem interval, age and gender (Table 2). When including more controls (*n* = 11) in the GLM analysis from our previously published dataset (Adorjan et al., 2017) there were no significant effects of variables (diagnosis, post-mortem interval, age, gender, position of sampling and time in PFA) on the density of NPY-ip neurons (Supplementary Table S7).

### Microglia Were Not Activated in the Caudate Nucleus in Patients With Schizophrenia

**FIGURE 5:**
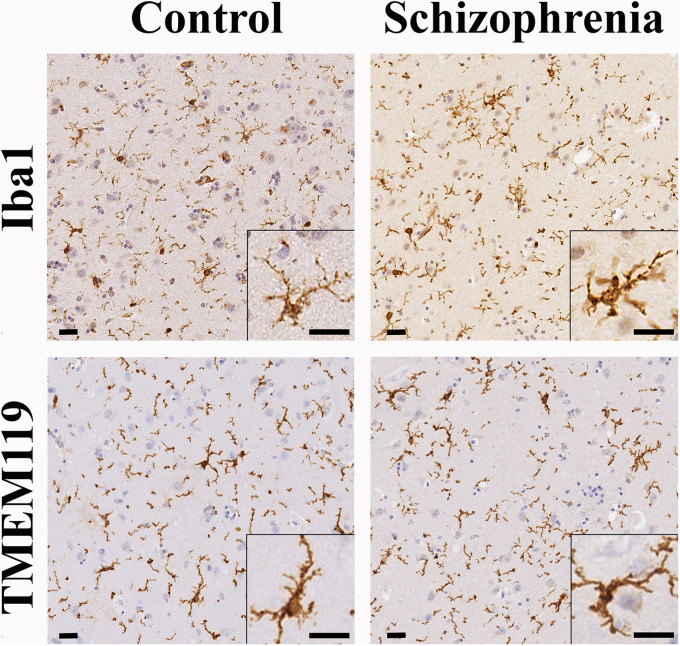
The distribution and morphology of ionized calcium-binding adapter molecule 1 (Iba1)-ip and TMEM119-ip microglia were similar in controls and cases with schizophrenia (SCH). The majority of microglia detected by Iba1 or TMEM119 had typical resting shape with small cell bodies and fine ramified processes both in controls and cases with SCH evaluated qualitatively. Scale bars: 20 μm.

In order to determine if the decrease in CR-ip density was associated with increased inflammation we carried out immunohistochemical analysis of the microglial markers Iba1 and TMEM119. Qualitative assessments of Iba1-ip and TMEM119-ip microglia morphology by three independent observers did not reveal microglial morphology differences between controls and cases with SCH (Figure 5). Microglia in the CN of both controls and subjects with SCH had typical resting morphology with small cell bodies and fine ramified processes. Amoeboid forms with larger cell bodies characteristic of activated microglia were occasionally seen in both diagnostic groups (Supplementary Figure S5). Quantitative analysis revealed no statistically significant differences between the control and SCH groups in the Iba1-ip stained area fraction (controls: 5.04% ± 0.42%, SCH: 3.66% ± 0.87%, *n* = 12, *p* = 0.164) or in the TMEM119-ip stained area fraction (controls: 3.32% ± 0.46%, SCH: 2.96% ± 0.72%, *n* = 12, *p* = 0.659), either. There was a significant positive correlation between Iba1-ip and TMEM119-ip stained area fractions measured in the CN (*r* = 0.688, *n* = 12, *p* = 0.013, Pearson’s correlation).

There were no significant effects of diagnosis or other variables (such as post-mortem interval, age, and gender) on the Iba1-ip and TMEM119-ip stained area fractions using GLM analysis (Table 2). For the Iba1-ip stained area fraction we could include 11 more controls in the GLM analysis from our previously published dataset (Adorjan et al., 2017) and found no significant effects of diagnosis, post-mortem interval, age, gender, position of sampling and time in PFA (Supplementary Table S7).

### The Density of Calretinin- and NPY-Immunopositive Neurons in the Dorsolateral Prefrontal Cortex

We were interested whether the observed changes in interneuronal composition of the CN in SCH were mirrored by alterations in the region primarily connected to the pre-commissural head of the CN, the DLPFC. In order to detect the density of CR- and NPY-ip cortical neurons, immunohistochemistry was carried out on samples from the medial frontal gyrus, a subregion of the DLPFC. The dominant CR-ip neuronal type in this region had elongated and bipolar morphology (Supplementary Figure S6) both in controls and patients with SCH. We did not find significant changes in the density of cortical CR-ip neurons (controls: 2,185 ± 130.04 cells/cm^2^, SCH: 2,264 ± 327.11 cells/cm^2^, *n* = 12, *p* = 0.985, Supplementary Figure S6 and Table 3). We did not find any morphological alterations or significant differences in the density of NPY-ip neurons, either (controls: 107 ± 18.16 cells/cm^2^, SCH: 119 ± 13.15 cells/cm^2^, *n* = 12, *p* = 0.582, Supplementary Figure S7 and Table 3). Finally, there were no significant differences found in the stained area fraction of Iba1 and TMEM119 in the DLPFC, suggesting no differences in microglial activation between controls and patients with SCH (Table 3).

**TABLE 3:** General Linear Model univariate analysis of CR, NPY densities and stained area fraction of Iba1 and TMEM119 in BA9 with PMI, age and gender as covariates.

	Diagnosis	PMI	Age	Gender
CR density	*p* = 0.876	*p* = 0.925	*p* = 0.405	*p* = 0.405
NPY density	*p* = 0.973	*p* = 0.774	*p* = 0.539	*p* = 0.376
Iba1 SAF	*p* = 0.854	*p* = 0.414	*p* = 0.219	*p* = 0.143
TMEM119	*p* = 0.719	*p* = 0.556	*p* = 0.323	*p* = 0.888

*CR, calretinin; BA9, Brodmann area 9; NPY, neuropeptide; Iba1, ionized calcium-binding adapter molecule 1; TMEM119, transmembrane protein 119.*

### The mRNA Levels of Calretinin, NPY, Iba1, and TMEM119 Were Not Significantly Altered in the Dorsolateral Prefrontal Cortex in Schizophrenia

Overall, we did not find any statistically significant changes regarding the transcript levels of CR and NPY in the DLPFC (Brodmann area 9) of patients with SCH (Table 4). However, there was a trend of lower CR mRNA expression (*p* = 0.061, Table 4). The transcript levels of Iba1 and TMEM119 were not statistically different between the two diagnostic groups (Table 4).

**TABLE 4:** General Linear Model univariate analysis of CR, NPY, Iba1, and TMEM119 mRNA levels in BA9 with PMI, age, and Gender as covariates.

	Diagnosis	PMI	Age	Gender
CR mRNA	*p* = 0.061	*p* = 0.369	*p* = 0.431	*p* = 0.752
NPY mRNA	*p* = 0.501	*p* = 0.605	*p* = 0.352	*p* = 0.975
Iba1 mRNA	*p* = 0.127	*p* = 0.770	*p* = 0.941	*p* = 0.243
TMEM119 mRNA	*p* = 0.391	*p* = 0.288	*p* = 0.243	*p* = 0.411

*CR, calretinin; BA9, Brodmann area 9; NPY, neuropeptide; Iba1, ionized calcium-binding adapter molecule 1; TMEM119, transmembrane protein 119.*

### Decreased Caudate Nucleus and Cortical Calretinin-Immunopositive Neuronal Densities Were Observed in Medication-Naïve Cases of Schizophrenia

We investigated if CR-ip neuronal densities in the CN or the DLPFC were correlated with medication history (Supplementary Table S8). There were two cases with no or minimal medication received in our study (#8 and #13, Supplementary Table S1). They provided the opportunity to test the hypothesis that decreased density of CR-ip neurons in the CN is associated with long-term anti-psychotic treatment but not observed in cases free of medication. However, this was not the case as the medication-naïve patients also had lower CR-ip densities in the CN (Figure 2) and in the DLPFC (Supplementary Figure S6). There was no correlation between the duration of anti-psychotic treatment and CR-ip densities in the CN or in the DLPFC (*r* = 0.103, *n* = 5; *r* = 0.086, *n* = 6, respectively, Spearmann’s rank correlation). These observations suggest that the decreased density of CR-ip neurons in SCH is independent of anti-psychotic treatment.

## Discussion

In this study, we demonstrated a strongly significant lower CR-ip CN interneuron density in SCH compared to controls, a major finding in the neuropathology of the condition. The importance of our result is underlined by the fact that no post-mortem neuronal subtype characterization has been carried out in the past 15 years concerning the CN in SCH. Nevertheless, our findings justify renewed interest in this effort.

In support of our work, several independent studies using different techniques identified CR-ip interneurons in the CN as possibly important in SCH. CN interneurons have been implicated in SCH by high throughput sequencing data and meta-analysis of common variant genomic results (Skene et al., 2018). Another robust meta-analysis of genome-wide association studies revealed 145 loci involved in SCH and CALB2 (the gene encoding CR) was a candidate locus (Pardiñas et al., 2018). Recent advances studying neuronal differentiation from SCH patient-specific iPSCs demonstrated that CR-ip interneurons exhibited impaired connectivity in cerebral organoids (Stachowiak et al., 2017). However, human iPSC-derived interneurons express CR very late in their maturation (> 20 weeks) which poses a major challenge in studying them (Nicholas et al., 2013). Nevertheless, human iPSCs have great potential to replicate patient-specific neurodevelopmental defects and to enable the discovery of underlying molecular mechanisms (Falk et al., 2016). We hope that our current and previous results on decreased CR-ip CN neurons in patients with SCH and ASD (Adorjan et al., 2017) will inspire future human iPSC studies employing not only cortical but also striatal differentiation assays in relation to these neuropsychiatric conditions.

There are several possible explanations for the observed lower density of CR-ip neurons in the CN. Firstly, it could theoretically be due to the loss of CR protein content without the actual loss of neurons. Such a scenario suggests the dysfunction of these neurons that can be caused by impedimental mechanisms at the transcriptional level or in post-translational modifications, or by mutations in the gene encoding CR (CALB2) which, as mentioned above, was recently identified as a candidate locus in SCH (Pardiñas et al., 2018). Deficit in CR protein would impair intracellular calcium-buffering which most probably impacts conductance and propagation of action potentials (Schurmans et al., 1997; Schiffmann et al., 1999). Secondly, the observed lower density may be caused by the actual loss of a substantial proportion of the CN neuronal population. This could be the result of various mechanisms such as degeneration of neurons in which CR protein had already been decreased – the loss of these neurons may be caused by long-term impairment of intracellular calcium-homeostasis. There may also be parallel mechanisms leading to intracellular calcium-overload and consequent neurodegeneration due to the impaired function of pre-synaptic scaffolding proteins in SCH (Gao and Penzes, 2015; Habela et al., 2016). Metabolic pathways selectively associated with CR-ip interneurons can be impaired in SCH, damaging this population. In fact, CR-ip interneurons are thought to be highly susceptible to metabolic insults (Hipólito-Reis et al., 2013) and to kainate toxicity (Lee et al., 2002), and thus compared to other interneuron populations, this susceptibility could lead to premature degeneration of CR-ip neurons. However, there is a line of earlier studies reporting that CR-ip neurons are actually selectively resistant to glutamatergic stress (Lukas and Jones, 1994; Möckel and Fischer, 1994). Future studies should re-assess and clarify the susceptibility of CR-ip interneurons to metabolic challenges by *in vitro* assays. Genome wide association studies identified a wide array of genetic mutations in synaptic scaffolding and ion-channel proteins in SCH (Fromer et al., 2014; Ripke et al., 2014; Pardiñas et al., 2018). Thus, another possibility is that inadequate astrocytic clearance of neurotransmitters from the synaptic cleft may exacerbate the metabolic strain/calcium overload in neuronal subpopulations where synaptic dysfunction had already been present. It should be emphasized that these pathological scenarios are not mutually exclusive and they could exacerbate one another. Future biochemical and transcriptomic studies at the single cell level are warranted to verify which of the aforementioned scenarios, or their combination, may cause the decreased density of CN CR-ip neurons in SCH.

There is a remarkable paucity of information on the electrophysiological properties, connectivity and function of CR-ip neurons in the CN. This may be because they are only 0.5% of neostriatal (caudate-putamen) neurons in rodents (Rymar et al., 2004) and therefore, they never received much attention. Interestingly, their proportion was dramatically increased during primate evolution and they account for 10% of all CN neurons in non-human primates (Deng et al., 2010), and human (Wu and Parent, 2000). The same evolutionary trend has been observed in the cerebral cortex (Barinka and Druga, 2010; Hladnik et al., 2014). Irrespective of their role in ASD and SCH the preponderance of these cells in our brains warrant their thorough investigation in non-human primate models and in human tissue. Our knowledge about CR-ip neuronal function is based on the rodent cerebral cortex where CR-ip neurons preferentially target other interneurons in layer 2/3 and pyramidal neurons in layer 5 (Gonchar and Burkhalter, 1999; Caputi et al., 2009). Consequently, if CR-ip neurons in the CN have broad postsynaptic preferences similar to their cortical counterparts (Meskenaite, 1997), they could innervate both aspiny interneurons and the principal output population of the CN, the medium spiny neurons (MSNs). Therefore they could exert both inhibitory and disinhibitory (excitatory) effects on the MSNs depending on their postsynaptic targets. It is equally important to investigate which neurons innervate the CN CR-ip interneurons. Excitatory cortical efferents, MSN collaterals or even other CN interneurons are amongst the several possible inputs that could profoundly affect CN CR-ip neuronal function. Electron microscopic studies in post-mortem human tissue and tract-tracing with rabies-virus trans-synaptic circuitry approaches in non-human primates may help address some of these important questions.

Post-mortem neuropathological research on neuropsychiatric diseases is hindered by the scarcity of brain tissue available. Enthusiasm for conducting post-mortem studies in SCH is further decreased by the dogma that long-term neuroleptic treatment may obscure neuropathological findings. Specifically relevant to our study, enlarged basal ganglia previously found in SCH may be related to medication (Harrison, 2000). An enlarged CN would cause a reduction in cell density even if cell numbers remained constant. However, recent advances in brain imaging examined 11,126 patients with SCH and 11,771 controls and accumulated strong evidence for the CN volume being unchanged in SCH (Haijma et al., 2013; van Erp et al., 2016). Also, there was no significant difference between CN volumes of medication-naïve patients and patients receiving anti-psychotic treatment except for the atypical anti-psychotic group (Haijma et al., 2013). Recently, there are studies reporting on the CN volume being unchanged (Kuo and Pogue-Geile, 2019; Takahashi et al., 2020) or even reduced in SCH (Akudjedu et al., 2020). Half of the SCH cases included in our study also received atypical anti-psychotic medication. However, analysis of cross-sectional areas of the CN did not indicate any volumetric increase in this group or in the group that only received typical anti-psychotics compared to control cases.

Although the hypothesis of CN structural alterations being due to anti-psychotic treatment was widely accepted in the past two decades, the concept was based on animal (mainly rodent) models (Harrison, 1999) and did not account for possible differences between rodents and primates in drug metabolism and effects. Here we did not find significant differences in the cross-sectional areas of the CN between patients with SCH and controls. In our study, we also showed that medication-naïve patients with SCH had decreased CR-ip interneuron density similar to patients on medication, suggesting that the CN interneuron phenotype is not caused by anti-psychotics. Similarly, there was no change in *in vivo* CN D2-receptor density between neuroleptic-naïve patients and patients receiving anti-psychotic treatment [median duration of treatment was 3.5 years (Lomeña et al., 2004)]. At the transcriptomic level there were no changes in striatal AMPA receptor subunits (GRIA1, GRIA2, GRIA3, GRIA4) expression levels between medicated and non-medicated groups of patients with SCH (Healy et al., 1998). However, at the cellular level there is a remarkable paucity of data on striatal neuronal populations in SCH or following chronic anti-psychotic treatment in non-human primates – suggesting more studies should focus on primate models. In summary, the long-held dogma on the enlargement of basal ganglia due to anti-psychotic medication (Harrison, 2000) should be revised, especially in the light of meta- and retrospective analyses which concluded that the majority of studies did not detect any significant volumetric changes regarding the CN following anti-psychotic medication (Ebdrup et al., 2013; Haijma et al., 2013).

The basal ganglia and the CN represent comparatively under-investigated areas in SCH research. Nevertheless, two studies focussed on the large choline-acetyltransferase-ip CN neurons in SCH which exhibit extensive overlap with the large CR-ip interneurons (Massouh et al., 2008). The densities of these cells were significantly decreased in the ventral CN in SCH (Holt et al., 1999; Holt et al., 2005). In our study we did not find a significant reduction in the large CR-ip interneuron density, neither throughout the CN nor in the ventral CN. Moreover, the analyzed area was substantially larger in our study (∼10 cm^2^) than in theirs (∼2 cm^2^; Holt et al., 2005). Our results also showed no statistically significant changes in the overall density of CR-ip interneurons or in CR mRNA levels in the cerebral cortex of patients with SCH, which is consistent with observations of several other groups (Daviss and Lewis, 1995; Reynolds and Beasley, 2001; Beasley et al., 2002; Cotter et al., 2002; Tooney and Chahl, 2004; Hashimoto et al., 2008; Fung et al., 2010). Furthermore, we did not find any statistically significant change in cortical NPY-ip neuron density in patents with SCH, similar to the work of Fung et al. (2010). However, others reported decreased NPY mRNA levels in the prefrontal cortex in SCH (Hashimoto et al., 2008; Morris et al., 2009; Mellios et al., 2009), suggesting that whereas NPY-ip neurons are not lost, expression of this marker may be decreased in SCH. Long-term anti-psychotic treatment may have more effects on synaptic structure than on neuronal density or size (Harrison, 1999). For example, in haloperidol treated rats synaptic rearrangements occurred without changes in total neuronal density or neuronal size (Benes et al., 1985). There were no changes in parvalbumin mRNA expression in the pre-frontal cortex of cynomolgus monkey after 9–12 months of haloperidol treatment (Hashimoto et al., 2003).

Although CR-ip and NPY-ip newborn neurons were reported in the adult human CN by Ernst et al. (2014), the exact origin of the newly generated interneurons is still unknown. The possible source(s) may be the subependymal zone of the lateral ventricle (Weissleder et al., 2019), striatal astrocytes (Duan et al., 2015; Nato et al., 2015), perivascular sources from striatal vessels (Stanton et al., 2015) or hitherto undiscovered migratory routes. Whereas hippocampal neurogenesis was found to be decreased in patients with SCH (Reif et al., 2006; Weissleder et al., 2019), a remaining question is whether subependymal zone neurogenesis is altered in patients with the condition. The subependymal zone of the lateral ventricle was examined in SCH, major depression and bipolar disorder and was not found to be histologically different from controls, however specific markers of neurogenesis were not used in that work (Comte et al., 2012). To further study the possibility of reduced CN neurogenesis in SCH an extensive panel of stem cell, proliferation and differentiation markers is needed. Our observation of the decreased density of CR-ip neurons being due to altered neurogenesis could be verified by multidisciplinary approaches in forthcoming investigations.

Some limitations of two-dimensional cell density analysis applied in our study should be discussed. Most importantly, differences in cell size between the investigated groups would lead to a source of error. However, average cell diameters and cross-sectional areas of CR-ip neurons were very similar in the two investigated groups. There is no indication in the literature of interneuronal volumes being altered in SCH. We do understand that the use of stereological cell counting would have returned unbiased results on cell numbers and three-dimensional density data of neuronal populations. However, due to the limited amount of tissue available from cases with SCH this approach was not feasible to us. Furthermore, the goal of our study was to identify quantifiable neuroanatomical differences between the diagnostic groups and not to generate three-dimensional ‘absolute’ measures for cell types. One can find more about the relative strengths and weaknesses of two-dimensional and three-dimensional densitometry approaches in the works of Benes and Lange (2001) and Williams et al. (2014).

According to stereological estimates, each human CN comprises approximately 50 million neurons (Wegiel et al., 2014). The functional importance of the CN is supported by the fact that most cerebral cortex areas [several billion neurons, (Pakkenberg and Gundersen, 1997)] project to it (Haber and Behrens, 2014; Jarbo and Verstynen, 2015). The pre-commissural CN preferentially receives inputs from cortical regions associated with SCH and ASD such as the DLPFC, ventromedial prefrontal cortex, anterior cingulate gyrus and superior temporal gyrus (Hirao et al., 2008; Haber and Behrens, 2014; Ciaramidaro et al., 2015). This makes the CN a perfect hub for information processing and selection where distant associative cortical fields converge. Moreover, the midbrain dopaminergic reward pathway and the mesolimbic system which have been heavily implicated in SCH also converge on neurons of the CN. It is also reasonable to propose that any significant alteration in CN neuronal composition may have major consequences in the function of associative and limbic modalities since abnormal signal gating in the CN can perturb function in various cortical regions via the direct and indirect striatal circuits (Nieuwenhuys et al., 2008; Prat et al., 2016). The impairment of these modalities could explain the core symptoms of SCH such as auditory/visual hallucinations and the decreased ability to infer mental states of others (theory of mind) in SCH.

A common etiology of SCH and ASD has been suggested by multiple approaches revealing shared genetic background (Habela et al., 2016; Wang et al., 2016), molecular mechanisms (Gao and Penzes, 2015; Canitano and Pallagrosi, 2017), and symptoms (Toal et al., 2009; Kästner et al., 2015) in these conditions. Especially, CN may be at the intersection of reward pathways (Kohls et al., 2014), of which impairments have been implicated in both SCH (Chung and Barch, 2016) and ASD (Dichter et al., 2012). The current study in SCH, together with our previous results on the decreased CR-ip CN phenotype in ASD (Adorjan et al., 2017) sheds light upon the similar neuropathology of these conditions manifested at the cellular level. These findings highlight a central role of the CN in these neuropsychiatric disorders and of CR-ip neurons as a population impaired in both SCH and ASD. Our results warrant further studies of the CN in SCH and ASD which have the potential to identify altered developmental programs in these conditions.

## Data Availability Statement

The raw data supporting the conclusions of this article will be made available by the authors, without undue reservation.

## Ethics Statement

The studies involving human participants were reviewed and approved by Oxford Brain Bank’s generic Research Ethics Committee approval (07/0606/85), Research Ethics Committee of the Hungarian Medical Research Council (45102-2/2016/EKU). The patients/participants provided their written informed consent to participate in this study.

## Author Contributions

IA and FGS designed the study and wrote the manuscript. IA and BS conducted IHC and qPCR experiments. IA, VF, and TT contributed to quantification. DV and SC provided support in statistical analyses. IA prepared the figures and tables. All authors read and approved the final manuscript.

## Conflict of Interest

The authors declare that the research was conducted in the absence of any commercial or financial relationships that could be construed as a potential conflict of interest.

## Footnotes

1 www.brainbank.nl

## Supplementary Material

Supplementary Figure 1 Position of coronal sections with the caudate nucleus (CN) analyzed in this study. Position of sampling of the CN from cases with SCH (shown in black) and CTR cases (shown in blue). Regarding case identifiers please refer to Table 1. Red line indicates the position of the anterior commissure.

Supplementary Figure 2 Scanned images with the caudate nucleus (CN) and dorsolateral prefrontal cortex (DLPFC) from CTR and schizophrenia (SCH). The regions of interests of the CN were outlined manually (green line) for cell measurement on whole-slide scans **(A,B)**. Regarding the DLPFC cortical columns (shown in various colors) were designated at equal distance from one another along the pial surface **(C,D)**. Scale bars: 5 mm **(A,B)**, 1 mm **(C,D)**.

Supplementary Figure 3 Correlation analyses between age and densities of calretinin-immunopositive (CR-ip) subpopulations in the caudate nucleus (CN) and dorsolateral prefrontal cortex (DLPFC). There was no statistically significant correlation (Pearson’s) regarding age and the densities of different CR-ip populations in the CN (small-CTR: *r* = 0.23, *p* = 0.46; small-schizophrenia (SCH): *r* = 0.57, *p* = 0.23; medium-CTR: *r* = 0.25, *p* = 0.63; medium-SCH: *p* = 0.67, *p* = 0.14; large-CTR: *r* = −0.18, *p* = 0.72; large- SCH: *r* = 0.45, *p* = 0.36) or even when these CR-ip subpopulations were taken together (CTR: *r* = 0.21, *p* = 0.68; SCH: *r* = 0.57, *p* = 0.22). When CTR and SCH groups were analyzed together, the statistical values were as it follows: small: *r* = 0.21, *p* = 0.50; medium; *r* = 0.35, *p* = 0.25; large: *r* = 0.15, *p* = 0.63; all subtypes: *r* = 0.23, *p* = 0.46. There was no statistically significant correlation between age and density of CR-ip neurons in the DLPFC, either (CTR: *r* = −0.01, *p* = 0.97; SCH: *r* = −0.26, *p* = 0.60, CTR and SCH combined: *r* = −0.19, *p* = 0.53). Red line: linear regression of CTR cases; blue line: linear regression of cases with SCH; black line: linear regression of cases with SCH and CTR combined.

Supplementary Figure 4 Correlation analysis between age and cross-sectional area of the caudate nucleus (CN). There was no statistically significant correlation between age and cross-sectional areas of the CN in the schizophrenia (SCH) group (*r* = 0.32, *p* = 0.59, Pearson’s correlation), in the CTR group (*r* = −0.37, *p* = 0.46, Pearson’s correlation), or even when these groups were merged (*r* = −0.14, *p* = 0.67, Pearson’s correlation). Red line: linear regression of CTR cases; blue line: linear regression of cases with SCH; black line: linear regression of cases with SCH and CTR combined.

Supplementary Figure 5 Amoeboid forms of microglia were occasionally seen in both CTR cases and cases with schizophrenia (SCH). Round, large cell bodies of microglia characteristic of activated cells were only rarely found in both diagnostic groups (arrows). Scale bars: 15 μm.

Supplementary Figure 6 The density of calretinin-immunopositive (CR-ip) neurons in the dorsolateral prefrontal cortex (DLPFC) was not significantly different in cases with schizophrenia (SCH) compared to controls. The predominant type of CR-ip neurons in the DLPFC had elongated and bipolar morphology in both controls and cases with SCH. Scale bars: 70 μm. Graph showing the number of CR-ip neurons per square cm in controls and subjects with SCH. ‘*x*’ labels a control case (#5) only received anti-psychotic medication as part of the palliative sedation treatment before euthanasia. ‘#’: these cases with schizophrenia (#8, #13) received minimal or no anti-psychotic medication.

Supplementary Figure 7 The density of neuropeptide Y-immunopositive (NPY-ip) neurons in the dorsolateral prefrontal cortex (DLPFC) was not significantly different in cases with SCH compared to controls. The predominant type of NPY-ip neurons in the DLPFC had multipolar morphology in both controls and cases with SCH. Scale bars: 40 μm. Graph showing the number of NPY-ip neurons per square cm in controls and subjects with SCH. ‘*x*’ labels a control case (#5) only received anti-psychotic medication as part of the palliative sedation treatment before euthanasia. ‘#’: these cases with schizophrenia (#8, #13) received minimal or no anti-psychotic medication.

Supplementary Table 1 Extended demographic characteristics of controls and patients with schizophrenia (SCH).

Supplementary Table 2 Average values of PMI, age, gender, and time in PFA in control and Schizophrenia (SCH) groups presented with the standard error of mean.

Supplementary Table 3 Cross-sectional areas of caudate nucleus (CN) and BA9 analyzed in the study with number of sections.

Supplementay Table 4 Parameters of the Aperio Positive Pixel Count Algorithm in the Iba1- and TMEM119-ip stained area fraction analysis.

Supplementary Table 5 Primer sequences used in the qPCR analysis.

Supplementary Table 6 General Linear Model univariate analysis of calretinin-immunopositive (CR-ip) densities in caudate nucleus (CN) with PMI, age, gender, position of sampling, and Time in PFA as covariates regarding our original cases (6 Sch and 6 Ctr) and 11 Ctr added from Adorjan et al. (2017).

Supplementary Table 7 General Linear Model univariate analysis of NPY density and Iba1+ stained area fraction in caudate nucleus (CN) with PMI, age, gender, position of sampling and time in PFA as covariates regarding our original cases (6 Sch and 6 Ctr) and 11 Ctr added from Adorjan et al. (2017).

Supplementary Table 8 Detailed list of anti-psychotic medications.
