# Supplementary figures and images for: Evidence for Decreased Density of Calretinin-Immunopositive Neurons in the Caudate Nucleus in Patients With Schizophrenia
Source: Front Neuroanat. 2020 Nov 13;14:581685. doi: 10.3389/fnana.2020.581685 (PMC7691639; doi:10.3389/fnana.2020.581685)

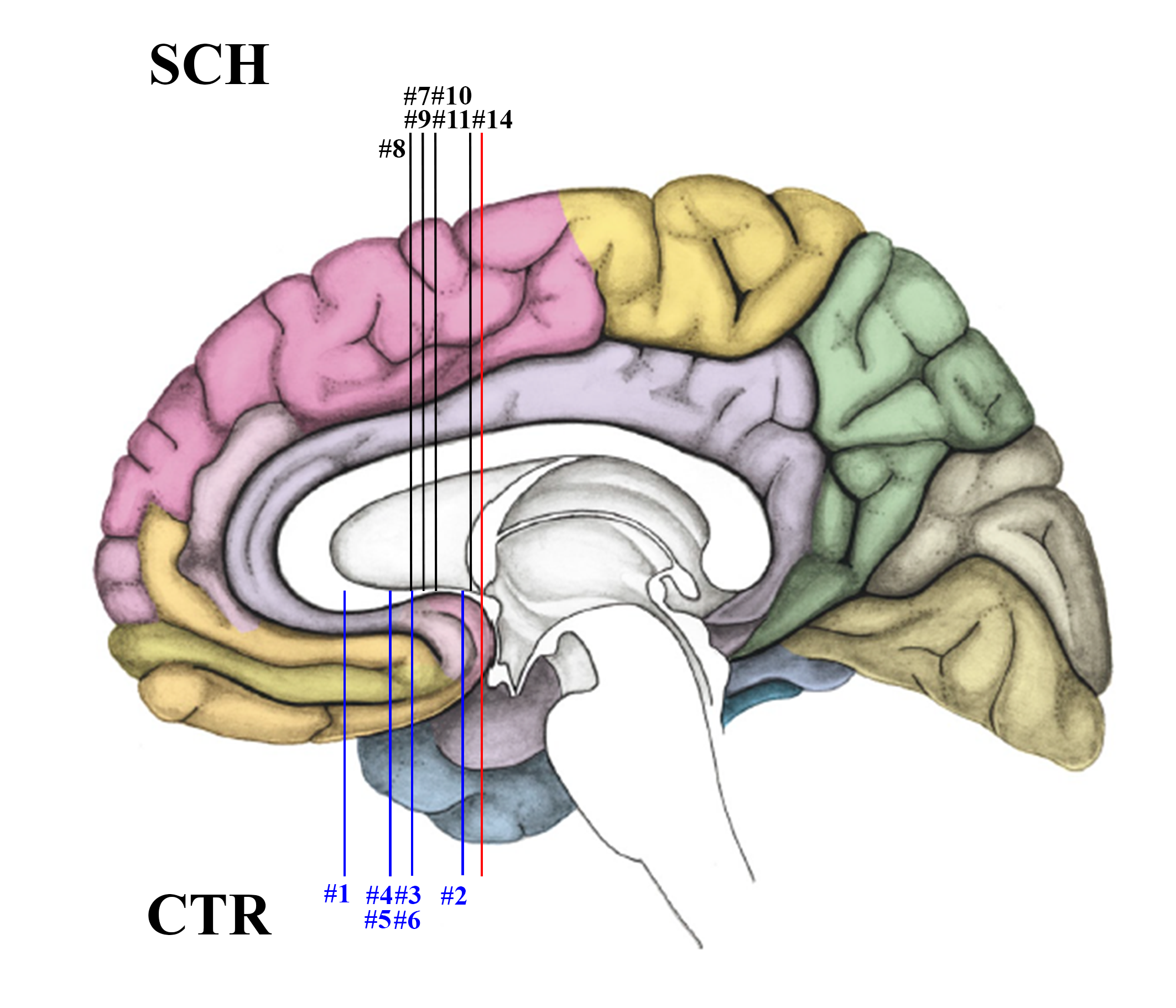

Supplement: Supplementary Figure 1 — Position of coronal sections with the caudate nucleus (CN) analyzed in this study. Position of sampling of the CN from cases with SCH (shown in black) and CTR cases (shown in blue). Regarding case identifiers please refer to Table 1. Red line indicates the position of the anterior commissure. [file Image_1.TIF]

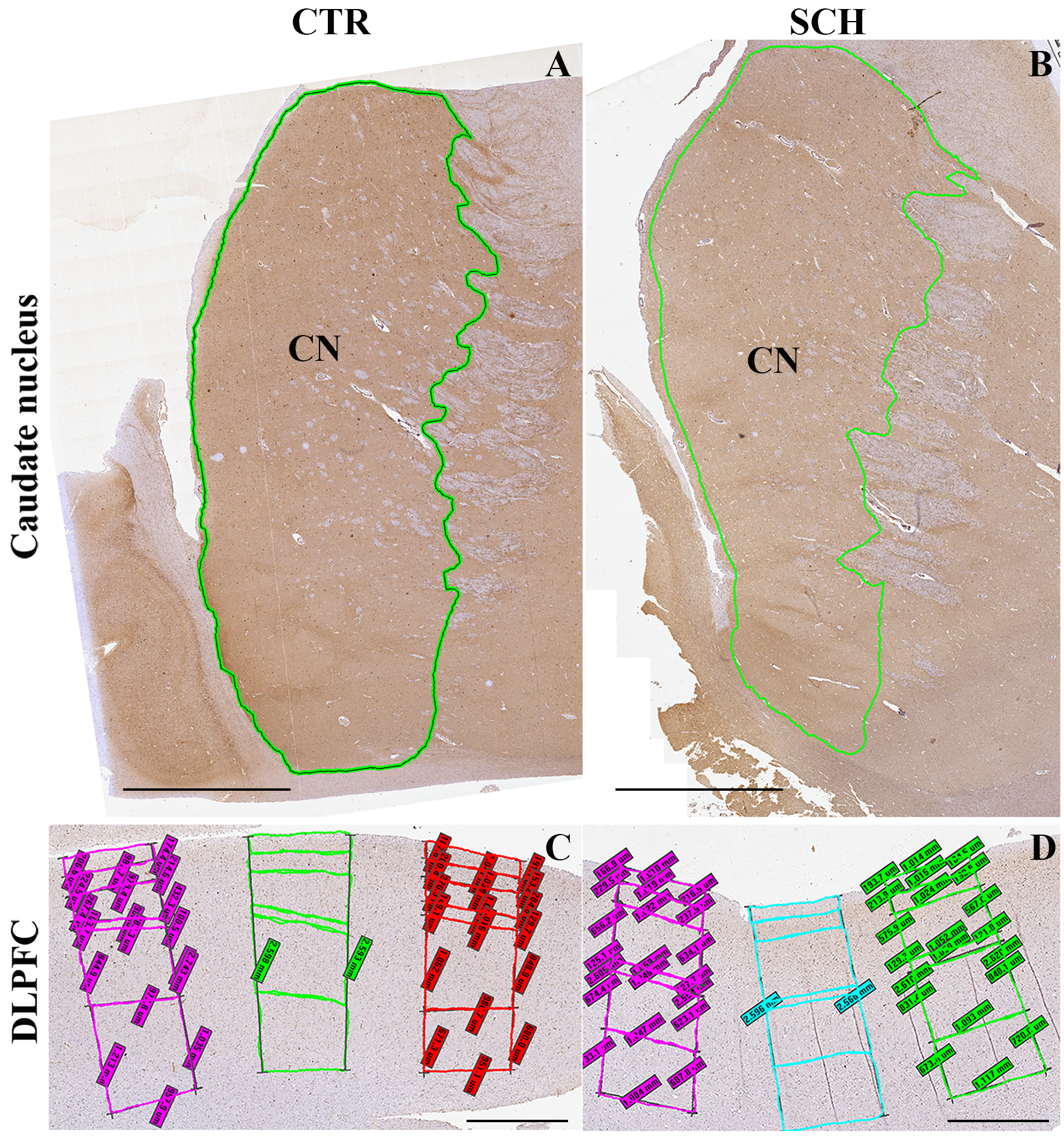

Supplement: Supplementary Figure 2 — Scanned images with the caudate nucleus (CN) and dorsolateral prefrontal cortex (DLPFC) from CTR and schizophrenia (SCH). The regions of interests of the CN were outlined manually (green line) for cell measurement on whole-slide scans (A,B). Regarding the DLPFC cortical columns (shown in various colors) were designated at equal distance from one another along the pial surface (C,D). Scale bars: 5 mm (A,B), 1 mm (C,D). [file Image_2.TIF]

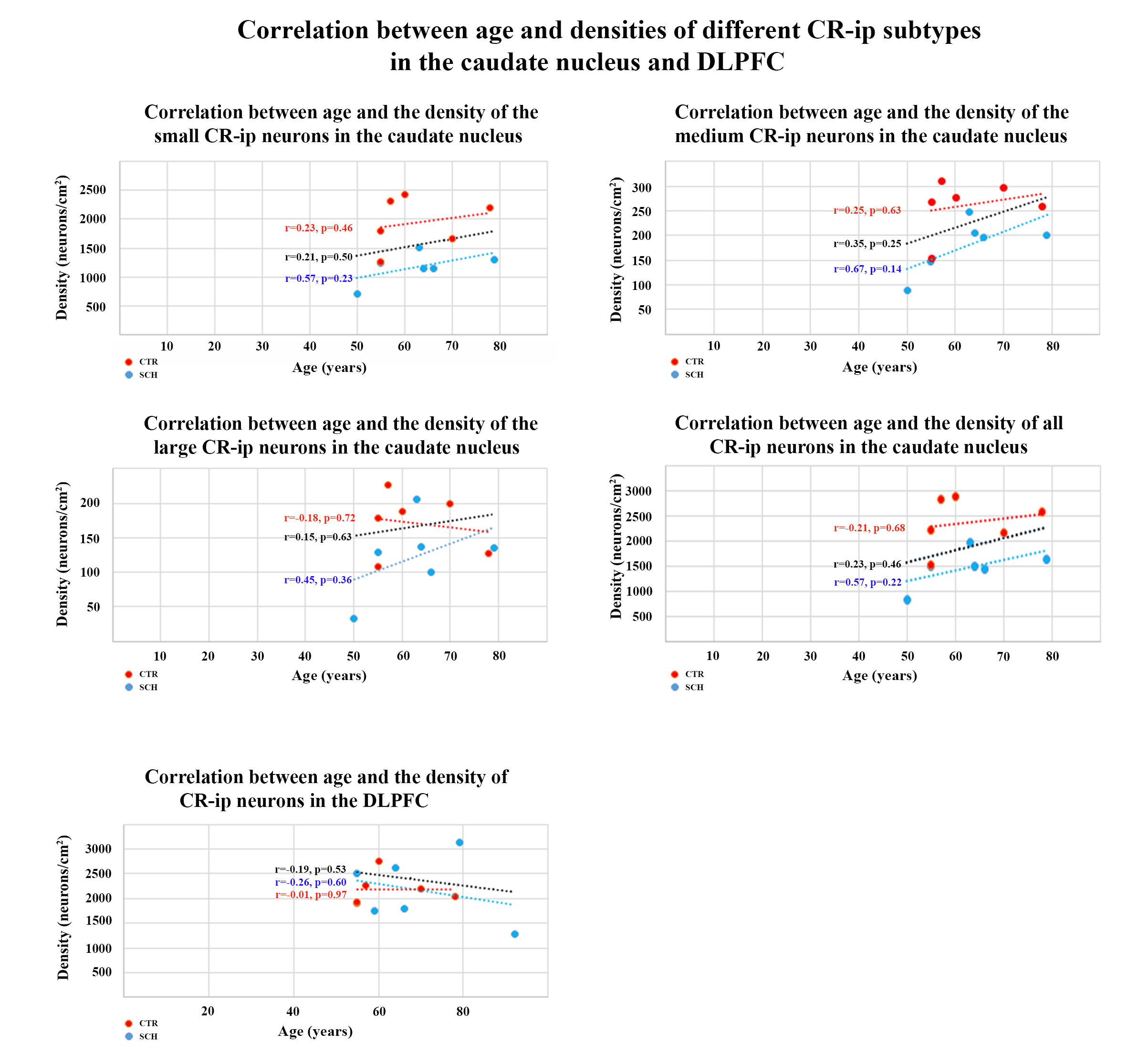

Supplement: Supplementary Figure 3 — Correlation analyses between age and densities of calretinin-immunopositive (CR-ip) subpopulations in the caudate nucleus (CN) and dorsolateral prefrontal cortex (DLPFC). There was no statistically significant correlation (Pearson’s) regarding age and the densities of different CR-ip populations in the CN (small-CTR: r = 0.23, p = 0.46; small-schizophrenia (SCH): r = 0.57, p = 0.23; medium-CTR: r = 0.25, p = 0.63; medium-SCH: p = 0.67, p = 0.14; large-CTR: r = −0.18, p = 0.72; large- SCH: r = 0.45, p = 0.36) or even when these CR-ip subpopulations were taken together (CTR: r = 0.21, p = 0.68; SCH: r = 0.57, p = 0.22). When CTR and SCH groups were analyzed together, the statistical values were as it follows: small: r = 0.21, p = 0.50; medium; r = 0.35, p = 0.25; large: r = 0.15, p = 0.63; all subtypes: r = 0.23, p = 0.46. There was no statistically significant correlation between age and density of CR-ip neurons in the DLPFC, either (CTR: r = −0.01, p = 0.97; SCH: r = −0.26, p = 0.60, CTR and SCH combined: r = −0.19, p = 0.53). Red line: linear regression of CTR cases; blue line: linear regression of cases with SCH; black line: linear regression of cases with SCH and CTR combined. [file Image_3.TIF]

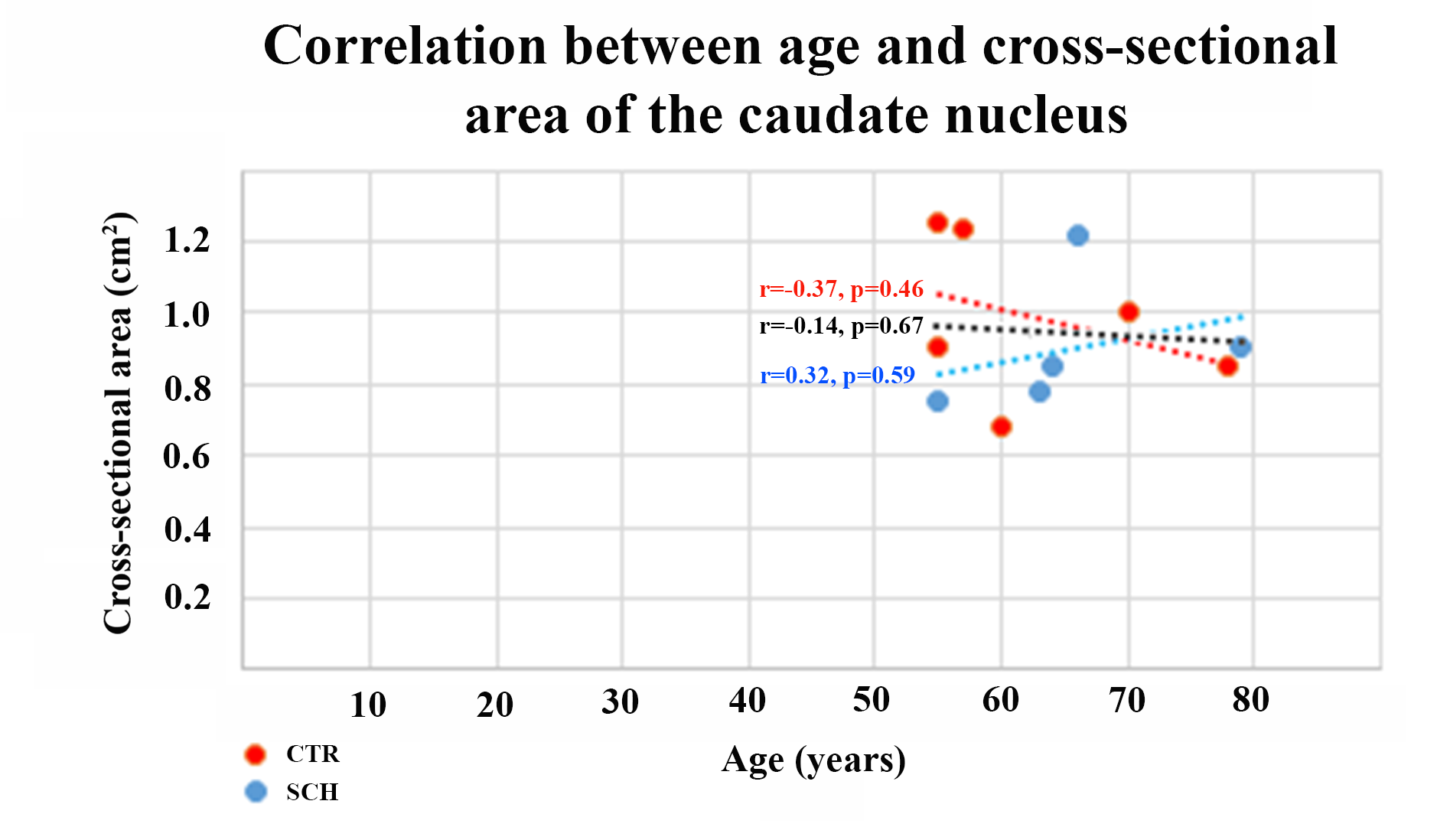

Supplement: Supplementary Figure 4 — Correlation analysis between age and cross-sectional area of the caudate nucleus (CN). There was no statistically significant correlation between age and cross-sectional areas of the CN in the schizophrenia (SCH) group (r = 0.32, p = 0.59, Pearson’s correlation), in the CTR group (r = −0.37, p = 0.46, Pearson’s correlation), or even when these groups were merged (r = −0.14, p = 0.67, Pearson’s correlation). Red line: linear regression of CTR cases; blue line: linear regression of cases with SCH; black line: linear regression of cases with SCH and CTR combined. [file Image_4.TIF]

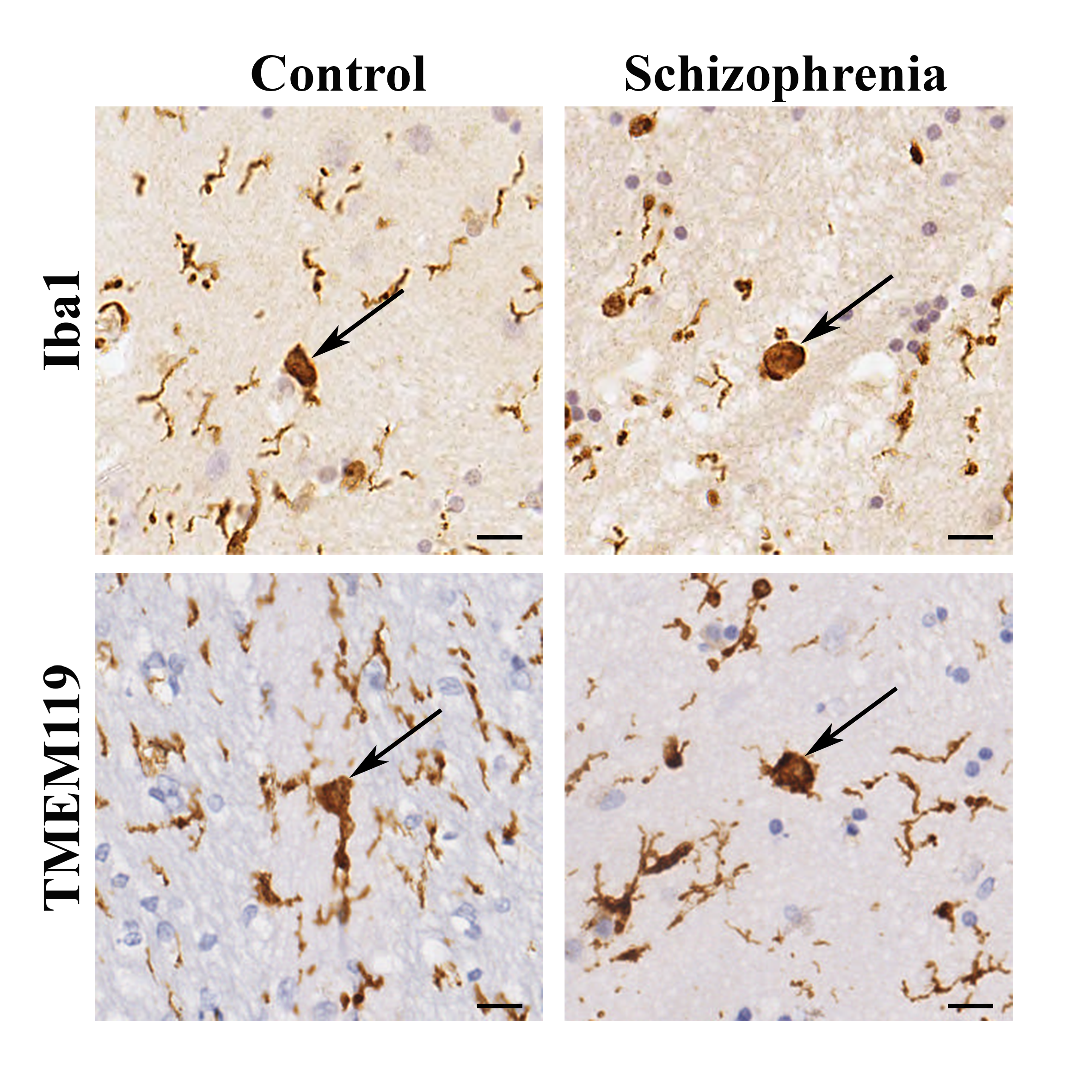

Supplement: Supplementary Figure 5 — Amoeboid forms of microglia were occasionally seen in both CTR cases and cases with schizophrenia (SCH). Round, large cell bodies of microglia characteristic of activated cells were only rarely found in both diagnostic groups (arrows). Scale bars: 15 μm. [file Image_5.TIF]

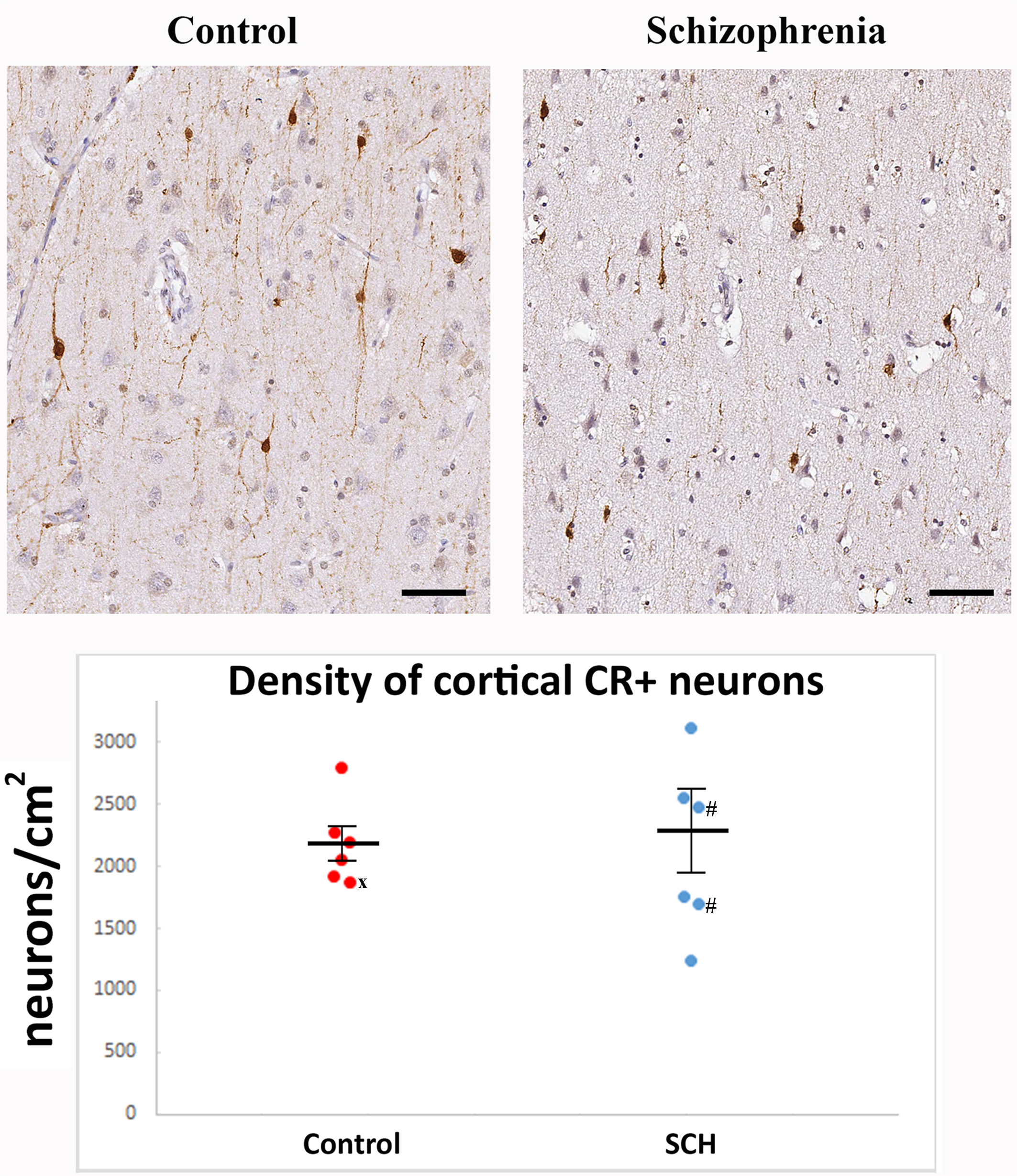

Supplement: Supplementary Figure 6 — The density of calretinin-immunopositive (CR-ip) neurons in the dorsolateral prefrontal cortex (DLPFC) was not significantly different in cases with schizophrenia (SCH) compared to controls. The predominant type of CR-ip neurons in the DLPFC had elongated and bipolar morphology in both controls and cases with SCH. Scale bars: 70 μm. Graph showing the number of CR-ip neurons per square cm in controls and subjects with SCH. ‘x’ labels a control case (#5) only received anti-psychotic medication as part of the palliative sedation treatment before euthanasia. ‘#’: these cases with schizophrenia (#8, #13) received minimal or no anti-psychotic medication. [file Image_6.TIF]

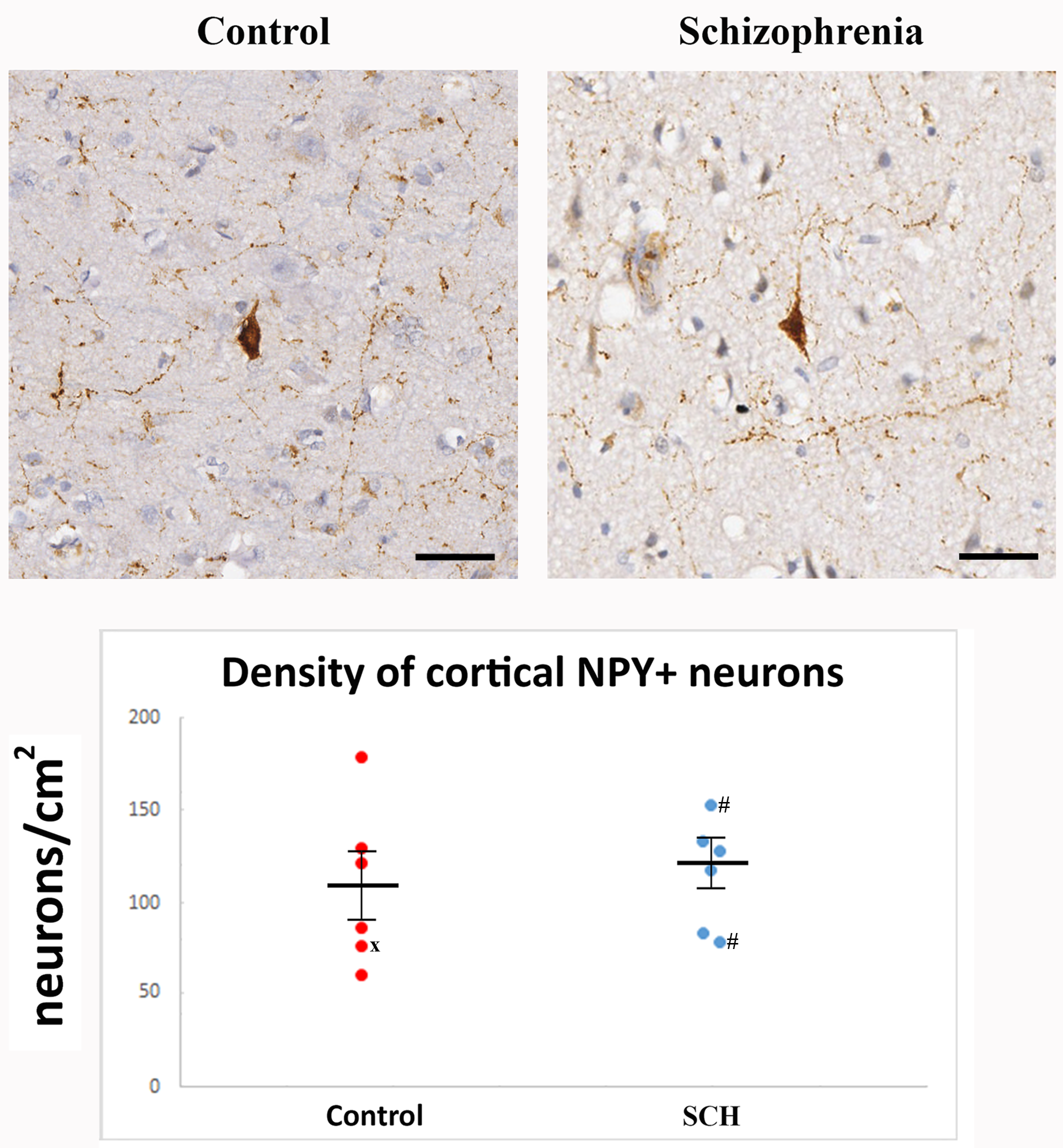

Supplement: Supplementary Figure 7 — The density of neuropeptide Y-immunopositive (NPY-ip) neurons in the dorsolateral prefrontal cortex (DLPFC) was not significantly different in cases with SCH compared to controls. The predominant type of NPY-ip neurons in the DLPFC had multipolar morphology in both controls and cases with SCH. Scale bars: 40 μm. Graph showing the number of NPY-ip neurons per square cm in controls and subjects with SCH. ‘x’ labels a control case (#5) only received anti-psychotic medication as part of the palliative sedation treatment before euthanasia. ‘#’: these cases with schizophrenia (#8, #13) received minimal or no anti-psychotic medication. [file Image_7.TIF]
