# Supplementary material for: Evidence for Decreased Density of Calretinin-Immunopositive Neurons in the Caudate Nucleus in Patients With Schizophrenia
Source: Front Neuroanat. 2020 Nov 13;14:581685. doi: 10.3389/fnana.2020.581685 (PMC7691639; doi:10.3389/fnana.2020.581685)
Supplement: Supplementary Table 1 — Extended demographic characteristics of controls and patients with schizophrenia (SCH). [file Table_1.pdf]

SUPPLEMENTARY TABLES

Table S1 Extended demographic characteristics of controls and patients with schizophrenia

Identifier	Diagnosis	Time in PFA (years)	Duration of anti-psychotic medication (years)	Type of anti-psychotic medication Typical=T Atypical=A	Source
#1	Control	0.1	0	none	NBB
#2	Control	0.1	0	none	NBB
#3	Control	0.1	0	none	NBB
#4	Control	0.1	0	none	NBB
#5	Control	0.1	0.16*	T	NBB
#6	Control	0.1	0	none	NBB
#7	SCH	0.1	30	T+A	NBB
#8	SCH	0.1	2	T	NBB
#9	SCH	0.1	40	A	NBB
#10	SCH	0.1	10	T+A	NBB
#11	SCH	0.1	10	T	NBB
#12	SCH	0.1	50	T+A	NBB
#13	SCH	0.1	0	none	NBB
#14	SCH	1	N/A	N/A	OBB

NBB=Netherlands Brain Bank, OBB=Oxford Brain Bank *only received anti-psychotic medication as part of the palliative sedation treatment before euthanasia

Table S2 Average values of PMI, Age, Gender and Time in PFA in control and SCH groups presented with the standard error of mean

	Age (years)	PMI (hours)	Gender (male=1, female=2)	Time in PFA (years)
**Control**	**62.5 ± 3.85**	**6.70 ± 0.46**	**1.66± 0.18**	**0.1± 0.01**
**Schizophrenia**	**66 ± 4.68**	**13.01 ± 3.42**	**1.63± 0.18**	**0.21± 0.11**
**Statistical comparison**	**p=0.579 Student’s t-test**	**p=0.109 Student’s t-test**	**p=0.950 Fischer’s exact test**	**p=0.350 Student’s t-test**

Table S3 Cross-sectional areas of CN and BA9 analysed in the study with number of sections.

Antigen - region	Control (area in cm^2)	Schizophrenia (area in cm^2)	Number of sections
**CR - CN**	**5.94**	**4.71**	**12**
**NPY - CN**	**5.66**	**4.41**	**12**
**Iba1 - CN**	**6.02**	**4.52**	**12**
**TMEM119 - CN**	**6.13**	**5.15**	**12**
**CR – BA9**	**0.51**	**0.50**	**12**

**NPY – BA9**	**0.53**	**0.56**	**12**
**Iba1 – BA9**	**0.53**	**0.50**	**12**
**TMEM119 – BA9**	**0.53**	**0.49**	**12**

CR=calretinin BA9=Brodmann area 9

Table S4 Parameters of the Aperio Positive Pixel Count Algorithm in the Iba1- and TMEM119-ip stained area fraction analysis.

**Hue Value**	**0.321**
**Hue Width**	**0.5**
**Color Saturation Threshold**	**0.04**
**Intensity Threshold Upper Limit of Weak Positive Pixels**	**255**
**Intensity Threshold Lower Limit of Weak Positive Pixels**	**255**
**Intensity Threshold Lower Limit of Medium Positive Pixels**	**150**
**Intensity Threshold Lower Limit of Strong Positive Pixels**	**0**
**Intensity Threshold of Negative Pixels**	**-1**

Table S5 Primer sequences used in the qPCR analysis

Gene	Forward primer	Reverse primer
**CALB2**	ACGACAAGGATAGAAGCGGC	ACAATCTCCAGGTCCTTGCG
**ACTB**	GGCATCCTCACCCTGAAGTA	AGCACTGTGTTGGCGTACAG
**GAPDH**	AATGAAGGGGTCATTGATGG	AAGGTGAAGGTCGGAGTCAA
**UBC**	GATCGCTGTGATCGTCACTT	TCTTTGCCTTGACATTCTCG
**PPIA**	ATGGTCAACCCCACCGTGTTCTTCG	CGTGTGAAGTCACCACCCTGACACA
**SNCA**	CAACAGTGGCTGAGAAGACCA	GCTCCTTCTTCATTCTTGCCCA
**IBA1**	GATGATGCTGGGCAAGAGAT	CCTTCAAATCAGGGCAACTC
**TMEM119**	CTTCCTGGATGGGATAGTGGAC	GCACAGACGATGAACATCAGC
**TBP**	CACGAACCACGGCACTGATT	TTTTCTTGCTGCCAGTCTGGAC
**SDHA**	TGGGAACAAGAGGGCATCTG	CCACCACTGCATCAAATTCATG
**NPY**	CCAGATACTACTCGGCGCTG	TGTCTCTGGGCTGGATCGTT

Table S6 General Linear Model univariate analysis of CR-ip densities in CN with PMI, Age, Gender, Position of sampling and Time in PFA as covariates regarding our original cases (6 Sch and 6 Ctr) and 11 Ctr added from Adorjan et al. 2017.

	**Diagnosis**	**PMI**	**Age**	**Gender**	**Position**	**Time in PFA**
**TotalCR density**	**p=0.002**	**p=0.091**	**p=0.284**	**p=0.144**	**p=0.730**	**p=0.334**
**SmallCR density**	**p=0.001**	**p=0.126**	**p=0.292**	**p=0.189**	**p=0.405**	**p=0.575**
**MediumCR density**	**p=0.185**	**p=0.069**	**p=0.325**	**p=0.223**	**p=0.067**	**p=0.110**
**LargeCR density**	**p=0.179**	**p=0.161**	**p=0.697**	**p=0.086**	**p=0.287**	**p=0.018**

CR=calretinin

Table S7 General Linear Model univariate analysis of NPY density and Iba1+ stained area fraction in CN with PMI, age, gender, position of sampling and time in PFA as covariates regarding our original cases (6 Sch and 6 Ctr) and 11 Ctr added from Adorjan et al. 2017.

	**Diagnosis**	**PMI**	**Age**	**Gender**	**Position**	**Time in PFA**
**NPY density**	**p=0.665**	**p=0.872**	**p=0.956**	**p=0.530**	**p=0.744**	**p=0.853**
**Iba1+ SAF**	**p=0.143**	**p=0.199**	**p=0.306**	**p=0.131**	**p=0.309**	**p=0.601**

Table S8 Detailed list of anti-psychotic medications

**Identifier**	**Anti-psychotic medication**	**Duration of treatment**
#5	haloperidol	2 months
#7	penfluridol<br>olanzapine	~25 years (1x20mg/week)<br>~2 years
#8	haloperidol	~2 years (4x1mg daily)
#9	olanzapine	~40 years (20mg/day)
#10	bromperidol<br>risperidone<br>clozapine<br>aripiprazole	~1 year (10mg/day)<br>~1 year (7mg/day)<br>~8 years (300mg/day)<br>~1 year (30mg/day)
#11	penfluridol<br>haloperidol	~10 years (20mg/week)<br>~1 year (1mg/day)
#12	pimozide<br>haloperidol<br>bromperidol<br>clozapine<br>risperidone	~20 years (5mg/day)<br>~7 years (2.5mg/day)<br>~5 years (100mg/3 weeks)<br>~10 years (2x100mg/day)<br>~3 years (2x2mg/day)
#13	no medication	0
#14	N/A	N/A
